# Antiviral Activity of Interferon Alpha-Inducible Protein 27 Against Hepatitis B Virus Gene Expression and Replication

**DOI:** 10.3389/fmicb.2021.656353

**Published:** 2021-03-31

**Authors:** Hafiz Ullah, Muhammad Sajid, Kun Yan, Jiangpeng Feng, Miao He, Muhammad Adnan Shereen, Qiaohong Li, Tianmo Xu, Ruidong Hao, Deyin Guo, Yu Chen, Limin Zhou, Li Zhou

**Affiliations:** ^1^State Key Laboratory of Virology, Modern Virology Research Center, College of Life Sciences, Wuhan University, Wuhan, China; ^2^MOE Key Laboratory of Tropical Disease Control, The Infection and Immunity Center, School of Medicine, Sun Yat-sen University, Shenzhen, China; ^3^Animal Biosafety Level III Laboratory at Center for Animal Experiment, Wuhan University, Wuhan, China; ^4^Department of Gynecology, Maternal and Child Health Hospital of Hubei Province, Tongji Medical College, Huazhong University of Science and Technology, Wuhan, China

**Keywords:** hepatitis B virus, IFI27, ISGs, antiviral activity, EnhII/Cp

## Abstract

Despite the availability of effective vaccines, hepatitis B virus (HBV) is still a major health issue, and approximately 350 million people have been chronically infected with HBV throughout the world. Interferons (IFNs) are the key molecules in the innate immune response that restrict several kinds of viral infections *via* the induction of hundreds of IFN-stimulated genes (ISGs). The objective of this study was to confirm if interferon alpha-inducible protein 27 (IFI27) as an ISG could inhibit HBV gene expression and DNA replication both in cell culture and in a mouse model. In human hepatoma cells, IFI27 was highly induced by the stimulation of IFN-alpha (IFN-α), and it potentiated the anti-HBV activity. The overexpression of IFI27 inhibited, while its silencing enhanced the HBV replication in HepG2 cell. However, the knocking out of IFI27 in HepG2 cells robustly increases the formation of viral DNA, RNA, and proteins. Detailed mechanistic analysis of the HBV genome showed that a sequence [nucleotide (nt) 1715–1815] of the EnhII/Cp promoter was solely responsible for viral inhibition. Similarly, the hydrodynamic injection of IFI27 expression constructs along with the HBV genome into mice resulted in a significant reduction in viral gene expression and DNA replication. In summary, our studies suggested that IFI27 contributed a vital role in HBV gene expression and replication and IFI27 may be a potential antiviral agent for the treatment of HBV.

## Introduction

Hepatitis B virus (HBV) is the main cause of chronic hepatitis B, hepatocellular carcinoma (HCC), and liver cirrhosis, and globally, it is considered the second major cause of cancer mortality (El-Serag, 2012). About two billion individuals are estimated to be infected by HBV. More than 650,000 people die annually due to HBV-related liver failure (World Health Organization, 2015).

Hepatitis B virus is a small enveloped virus, which belongs to the prototype member of the Hepadnaviridae family, having partially double-stranded circular DNA with a 3.2-kb genome (Robinson and Lutwick, 1976). After infection, HBV binds to sodium taurocholate co-transporting polypeptide (NTCP) on hepatocytes eliciting HBV entry and the subsequent transfer of nucleocapsid into the cytoplasm (Yan et al., 2012; Ni et al., 2014). The HBV genome is uncoated in the host cells’ cytoplasm and then transported to the cell nucleus, where relaxed circular DNA (rcDNA) of the virus is transformed into covalently closed circular DNA (cccDNA) (Qi et al., 2016). The cccDNA serves as a template for virus transcripts, such as 3.5 [pregenomic RNA (pgRNA], 2.4, 2.1, and 0.7 kb messenger RNAs (mRNAs). The viral transcription process is controlled by four promoters (core, preS1, preS2/S, and X promoters) and two liver-specific enhancers (EnhI and EnhII). EnhI and EnhII endow liver-specific expression of viral gene products (Yee, 1989). HBV mRNAs are used for encoding seven types of proteins: 3.5 kb mRNA for the HBV polymerase (pol); secreted HBcAg and HBeAg; 2.4 and 2.1 kb mRNAs for large, middle, and small surface proteins (L, M, and S HBsAg); and 0.7 kb mRNA for the HBx protein (Liang, 2009). The core promoter is involved in the formation of pgRNA, so the regulation of this promoter is very important in the life cycle of HBV (Kramvis and Kew, 1999). Therefore, transcription silencing of the HBV core promoter may be a pretty good approach for HBV. It was reported previously that liver-enriched transcription factors (LETFs), such as hepatocyte nuclear factor-4 alpha (HNF4α), retinoid X receptor alpha (RXRα), peroxisome proliferator-activated receptor alpha (PPARα), and farnesoid X receptor alpha (FXRα), play a key role in regulating the activity of the core promoter and contribute to the regulation of HBV transcription and replication. Therefore, targeting LETFs can help control HBV infection (Schrem et al., 2002; Chen et al., 2012).

Interferon (IFN) is part of the initial response to invade infectious agents and to induce the expression of dozens of IFN-stimulated genes (ISGs) (Schoggins et al., 2015). Type I IFNs are key contributors that have long been recognized as an effective antiviral response (Murira and Lamarre, 2016). Type I IFN binds to the cell surface receptor (IFNARI/2) and initiates a signaling cascade through the Janus kinase signal transducer and activators of transcription (JAK-STAT) pathway, resulting in the induction of hundreds of ISGs (Ivashkiv and Donlin, 2014). According to the type of receptor to which IFNs bind, human IFNs are generally divided into type I, type II, and type III (Isaacs and Lindenmann, 1957; Sheppard et al., 2003; Vilcek, 2006). Interferon alpha (IFN-α) is a kind of type I IFN with antiviral activity: as a host cytokine, it was the first remedy approved for the treatment of HBV infection, which has antiviral effects (Baron et al., 1980). Type I IFNs (IFN-α, IFN-β, IFN-ε, IFN-κ, and IFN-ω) are presently approved for the treatment of chronic hepatitis B (CHB) (De Andrea et al., 2002), and IFN-α has been testified to restrict HBV gene expression and replication in other systems *in vitro* (Wieland et al., 2003; Pollicino et al., 2013). IFN-α and pegylated IFN-α have been approved for the treatment of CHB (De Andrea et al., 2002; Lee and Baldridge, 2017). Despite severe side effects, type I IFN treatment remains an antiviral option for treating CHB. Due to the limitations of IFN-α, it was slightly improved with combination therapy, such as with entecavir (ETV) or tenofovir (Zhuang, 2012).

Interferon alpha-inducible protein 27 (IFI27) or ISG12a belongs to the IFI6/IFI27 family, which comprises a conserved 80 amino acid motif called the ISG12 motif (Parker and Porter, 2004; Cheriyath et al., 2011). IFI27 or ISG12a was first named as interferon alpha-inducing protein 27 (p27) in estradiol-treated MCF7 human breast carcinoma cells. An additional study showed that the *IFI27* gene was located in band q32 of human chromosome 14 and was greatly inducible by IFN-α in numerous human cell lines (Rasmussen et al., 1993). *IFI27* or *ISG12a* (MW 11.5 kDa) and *6-16* (MW 12.9 kDa) are type I ISGs encoding small hydrophobic proteins. These proteins have 36% overall amino acid similarity and 49% identity over an ∼80 amino acid length. Both ISGs are regulated by type I IFNs in various types of cell lines (Kelly et al., 1986; Porter et al., 1988). Humans have four members of the IFI6/IFI27 family, including *ISG12a*, *ISG12b*, and *ISG12c* genes, while mice have only three family members, containing the *ISG12a*, *ISG12b1*, and *ISG12b2* genes, and lack the *IFI6* gene ortholog (Labrada et al., 2002; Parker and Porter, 2004). Interestingly, type I IFN highly induced the human ISG12a (IFI27), whereas the ISG12b and ISG12c are not inducible by IFN (Liu et al., 2007). In order to clarify the antiviral mechanism of cytokines, 36 ISGs along with IFI27, which are highly induced in liver cells, were tested for their ability to inhibit HBV replication when overexpressed in human hepatoma cells (Mao et al., 2011). The genomic microarray analyses showed that HepG2.2.15 cells transfected with siRNA expression vectors (siRNA-1 and siRNA-7) changed the expression of 18 genes, 10 of which were immune response-related genes and 9 of them (*IFIT1*, *MDA5*, *STAT1*, *G1P2*, *IFI27*, *IFITM1*, *OAS1*, *G1P3*, and *ISGF3G*) were ISGs. These genes may be involved in the interaction of HBV with the host cells and cellular genes in response to HBV (Guo et al., 2005). Only a few other studies have assessed the biological activity of the IFI27 protein. It was reported in a previous study that high basal IFI27 or ISG12a may inhibit Newcastle disease virus (NDV) replication and oncolysis, whereas low basal IFI27 may allow sufficient NDV replication for induction of IFI27 (Liu et al., 2014). IFI27, through its non-apoptotic antiviral activity, targets viral NS5A protein through a proteasome-dependent pathway in HCV-infected cells (Xue et al., 2016). Murine IFI27 exhibited antiviral activities on West Nile virus (WNV) and murine hepatitis virus (MHV) with unknown mechanisms (Cho et al., 2013; Lucas et al., 2016). This suggests that IFI27 may play a vital role in innate antiviral immunity that could provide a critical clue to explore the inhibitory mechanisms of innate immunity to HBV infection.

In this study, we investigated a detailed analysis and mechanism of the inhibitory effect of IFI27 on HBV replication and transcription in human hepatoma cells as well as in a mouse model system. The overexpression of IFI27 inhibits HBV replication and transcription, whereas knockdown and knocking out in HepG2 cells enhances HBV replication and transcription. Likewise, the introduction of IFI27 into mice showed a significant reduction in HBV DNA replication and gene expression. Importantly, it was revealed that IFI27 could inhibit HBV through inhibition of EnhII/Cp promoter activity, which plays a vital role in HBV replication. Notably, our data suggest that IFI27 may aid as an effective therapeutic option for the treatment of HBV in the future.

## Materials and Methods

### Plasmids

The pHBV1.3 plasmid (genotype D, GenBank accession number V01460.1) was constructed as previously reported (He et al., 2016). The plasmid expressing N-terminally hemagglutinin (HA)-tagged-IFI27 fragment was PCR amplified and inserted into *Eco*RI and *Xho*l sites of the pCAGGS vector. The construction of the reporter plasmids pGL3-SP1-Luc, pGL3-SP2-Luc, pGL3-EnhII/Cp-Luc, and pGL3-EnhI/Xp-Luc was followed as reported previously (Hao et al., 2015). Two IFI27 short hairpin RNAs (shRNAs) and control or non-targeting shRNA (shControl) containing enhanced green fluorescence protein (eGFP) were inserted into pLKO-1-puro. The targets for the shRNAs are as follows: shControl: 5′-GCAGAAGAACGGC ATCAAG-3′, ShIFI27-4: 5′-CTCCGGATTGACCAAGTTCAT-3′, and ShIFI27-5: 5′-CCCTGCAGAGAAGAGAACCAT-3′.

### Animal Work Ethical Approval

All animal experiments were performed following the Guide for the Care and Use of Laboratory Animals of the National Institutes of Health. Mice were housed under specific pathogen-free (SPF) conditions in individually ventilated cages. The protocol was approved by the Institutional Animal Care and Use Committee of Wuhan University (project license WDSKY0201302).

### Cell Culture and Transfection

HepG2, Huh7 cells, HepG2.2.15, and human embryonic kidney 293T (HEK-293T) cells were cultivated in Dulbecco’s modified Eagle’s medium (complete DMEM) comprising 10% fetal bovine serum (FBS), 100 U/ml penicillin, and 100 mg/ml streptomycin. Transfection of cells was carried out with Lipofectamine 3000 (Invitrogen) according to the manufacturer’s instructions. The cells were retained at 37°C in a humidified 5% CO_2_ atmosphere.

### CRISPR/Cas9 Gene-Editing Technique

For the generation of HepG2 cells in which IFI27 was knocked out (IFI27-KO), the gene-editing technology CRISPR/Cas9 was used. The gRNA targeting Cas9 to IFI27 gene was cloned. HEK-293T cells were co-transfected with Lenti-IFI27-gRNA-CRISPR-Cas9 plasmid together with the packaging vector pMD2.G and psPAX using Neofect reagent (Neofect Biotech, Beijing, China), and the lentiviruses were harvested after 48 and 72 h. These lentiviral particles were then transduced into HepG2 cells in the presence of polybrene (8 μg/ml), with the subsequent selection of puromycin (2 μg/ml). Cells were plated in a 96-well plate at ∼1 cell per well to get a single clone. Western blotting for individual clones was used to detect the expression of IFI27. KO cells were also confirmed by sequencing the targeted loci. The sequence of gRNA is as follows: 5′-CTCTGCCGTAGTTTTGCCCC-3′.

### HBV DNA Extraction and Analysis

The extraction technique of HBV DNA from intracellular core particles was adopted from a previously described method with a few modifications (He et al., 2016). In brief, HepG2 cells were co-transfected with pHBV1.3 and pSV-β-gal along with pCAGGS-HA-IFI27 or its control vector (pCAGGS) in the quantities as shown in the related figures. The HepG2 cells were lysed in NP-40 lysis buffer [50 mM Tris–HCl (pH 7.0) and 0.5% NP-40] at 4°C after 96 h post-transfection and then centrifuged at 13,000 rpm. The supernatants were collected and digested with RNase A and DNase I (Thermo Fisher Scientific) in the presence of DNase I buffer at 37°C for 2 h, DNase I was inactivated subsequently at 65°C for 20 min in the presence of 10 mM EDTA, and then proteinase K was used to digest the protein along with 1% SDS overnight at 55°C. Finally, the digested samples were extracted with phenol:chloroform. After that, DNA samples were precipitated with ethanol and then resolved in 30 μl Tris-EDTA (TE) buffer. The viral DNA was then subjected to qPCR. The extracellular encapsidated DNA of HBV from supernatant or sera was extracted following a previously described protocol (Tian et al., 2011; Hao et al., 2015). The HBV replicative intermediate and extracellular HBV DNA were then analyzed by qPCR using primers as mentioned in Table 1.

**TABLE 1:** The sequence of primers used in this study.

Name	Sequence (5′–3′)	Method used
IFI27-F	GCCTCTGCTCTCACCTCATC	PCR cloning
IFI27-R	ATCTTGGCTGCTATGGAGGA	
RCCCS	CTCGTGGTGGACTTCTCTC	qPCR
RCCCAS	CTGCAGGATGAAGAGGAA	
Total HBV mRNA-F	GAGTGCTGTATGGTGAGGTG	qRT-PCR
Total HBV mRNA-R	TTTGGGGCATGGACATTGAC	
hGAPDH-F	CCACCCATGGCAAATTCCATGGCA	qRT-PCR
hGAPDH-R	TCTAGACGGCAGGTCAGGTCCACC	
3.5 kb mRNA-F	GCCTTAGAGTCTCCTGAGCA	qRT-PCR
3.5 kb mRNA-R	GAGGGAGTTCTTCTTCTAGG	
EnhII/CP-F	ACTGTTTGTTTAAAGACTGGGAG	CHIP PCR
EnhII/CP-R	GGTGCTGGTGCGCAGACCAATTTA	
Mice GAPDH-F	ATGGTGAAGGTCGGTGTGAA	qRT-PCR
Mice GAPDH-R	CGCTCCTGGAAGATGGTGAT	
IFI27Q-F	TCACCTCATCAGCAGTGACC	qRT-PCR
IFI27Q-R	ATCTTGGCTGCTATGGAGGA	

### Western Blot

HepG2 cells were lysed in cold lysis buffer [50 mM Tris–HCl, 100 mM NaCl (pH 8.0), 5 mM EDTA, 1% SDS) and a cocktail (protease inhibitor) was added. The samples were separated on SDS polyacrylamide gel electrophoresis (PAGE) and then transferred to nitrocellulose membrane. The membrane was incubated for 1 h in 5% non-fat dried skim milk to block non-specific binding. Following the membrane incubation at 4°C with target-specific primary antibodies such as rabbit anti-IFI27 (Biorbyt, Catalog number: orb337785) or anti-Ha-IFI27 (Abcam Trading Company, Shanghai, China) overnight. The membrane was incubated with a secondary antibody for 1 h and the result was normalized to reference internal control β-actin (ABclonal, Woburn, MA, United States). The signal of the western blot was observed with an enhanced chemiluminescence (ECL) substrate (Millipore, Billerica, MA, United States).

### Extraction and Analysis of RNA

The total RNA from HepG2 cells and tissue from mice liver was isolated using TRIzol reagent (Invitrogen, Carlsbad, CA, United States). The cDNA was synthesized using Reverse Transcription Kit (Takara Biomedical Technology). PCR reactions were prepared using SYBR Green Fast qPCR Master Mix Kit (Yeasen Biotechnology, Shanghai). The mRNA of the housekeeping glycolysis gene glyceraldehyde-3-phosphate dehydrogenase (*GAPDH*) functioned as an endogenous standard. The ΔΔCt method was used and all the primers used in qRT-PCR are mentioned in Table 1.

To perform the northern blot, 4 μg of RNA samples were taken and then resolved on MOPS-buffered 1.5% agarose gel comprising 2.2 M formaldehyde and transferred to a nylon membrane (GE Healthcare). The hybridization was done adopting the manufacturer’s instructions containing the DIG-labeled probe. The probe was generated with a DIG probe synthesis kit (Roche, Germany). The rRNAs (28S and 18S) act as an internal control to detect the quantity of total RNA.

### Analysis of Secretory Hepatitis B Antigen

The levels of HBsAg and HBeAg antigen in culture supernatants of transfected HepG2 cells or mice serum were assessed using an enzyme-linked immunosorbent assay (ELISA) according to the manufacturer’s protocol (Kehua District, Shanghai). The activity of β-galactosidase was used to normalize the values in cell lysates and measured by a Beta-Glo kit (Promega).

### Dual-Luciferase Assays (Reporter Assays)

HepG2 cells plated in a 24-well plate were transfected with HBV reporter plasmids (200 ng) along with an indicated amount of pCAGGS-HA-IFI27 plasmid (250 ng) or pCAGGS (control vector), and the pRL-TK plasmid (50 ng) was used for the control of transfection efficiency. At 48 h post-transfection, the cells were lysed and subjected to luciferase activity assay using the Dual-Glo system (Promega, Madison, WI, United States).

### Chromatin Immunoprecipitation Assay

For HBV promoter analysis, chromatin immunoprecipitation (ChIP) assay was performed using the standard protocol of the ChIP Assay kit (Beyotime, Biotech, Catalog number: P2078). Briefly, HepG2 cells were grown to confluency in 10-cm dishes and transfected with HBV1.3 with IFI27 or control vector (pCAGGS). At 72 h post-transfection, the cells were cross-linked with formaldehyde for 10 min at 37°C, followed by neutralization using 125 mM glycine. The cells were then lysed with cold lysis buffer (50 mM Tris–HCl, 150 mM NaCl, pH 8.0, 10 mM EDTA, 1% SDS) in combination with a cocktail (protein inhibitor). Then, the lysates were sonicated by three pulses for 15 s on ice to break the genome into 200–1,000 bp in size. The antibodies used for immunoprecipitation were the anti-HA antibody (Abcam, Catalog number: ab9110) at 4°C overnight. Normal mouse IgG and anti-RNA polymerase II were used as negative and positive controls, respectively. The precipitate was then washed, and immunoprecipitated DNA fragments were extracted with phenol–chloroform extraction. The immunoprecipitated DNA fragments were amplified with PCR using EnhII/Cp (nt 1715–1815) primers shown in Table 1.

### Hydrodynamics-Based Transfection in Mice

To test HBV replication *in vivo*, 6–8-week-old male mice (C57BL/6) were used. We randomly divided a total of 10 mice into two groups (five mice each). The replication-competent vector pHBV1.3 (10 μg) and pSV-β-gal (5 μg) were hydrodynamically co-delivered together with pCAGGS-HA-IFI27 expression constructs or empty vector pCAGGS (20 μg) into the tail vein of mice within 8 s in a volume of saline equivalent to 10% of the mouse body weight. These mice were sacrificed after 4 days post-injection and mice livers and blood were processed for analysis. The mice sera were collected and examined for analysis of HBsAg, HBeAg, and HBV DNA. Western blot was performed with a small slice of mice liver to detect IFI27 and HA-tag protein. For HBV RNA analysis, a small piece of liver tissue was homogenized in TRIzol reagent to purify and extract total RNA. Liver tissues were also collected to analyze HBV core antigen expression by using immunohistochemical staining.

### Statistical Data

All experiments were repeated at least three times. The results are presented as means ± SD unless stated otherwise. The statistical significant differences were determined by using one-way ANOVA analysis with multiple comparison test and independent Student’s *t*-test. Statistical analyses were achieved using the Prism 8 software (GraphPad Software Inc., San Diego, CA, United States). A *P* ≤ 0.05 was considered statistically significant.

## Results

### IFN-α Induces IFI27 Expression and the Role of IFI27 in IFN-Elicited Anti-HBV Response

**FIGURE 1:**
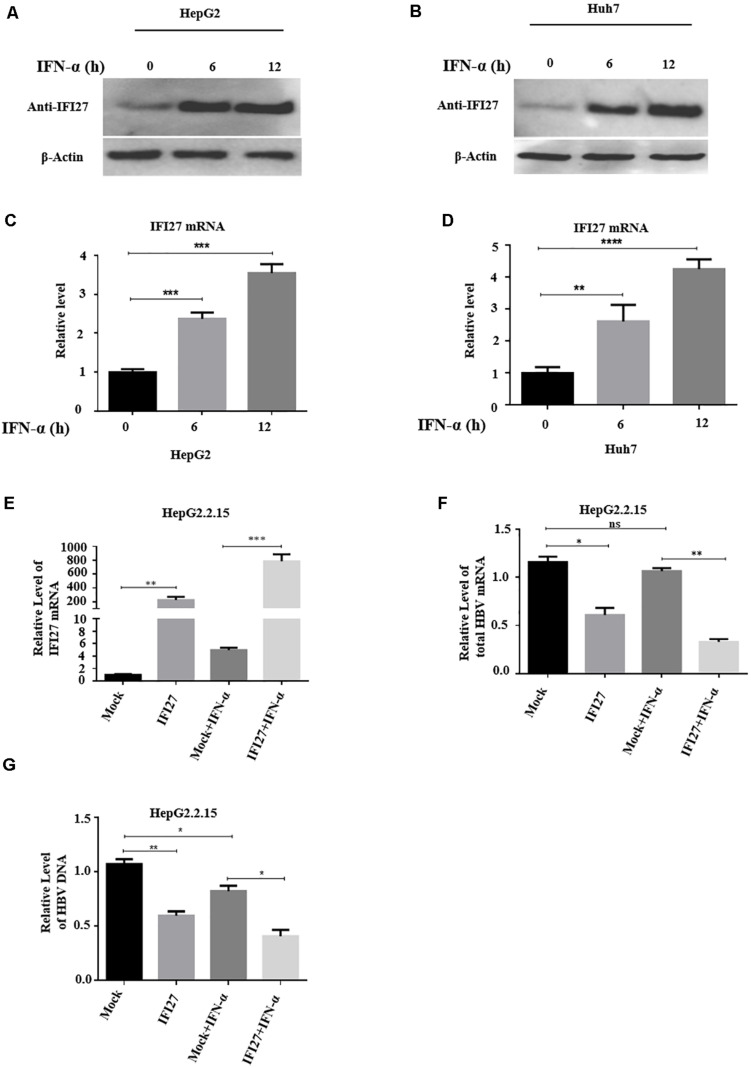
Interferon alpha (IFN-α) induces interferon alpha-inducible protein 27 (IFI27) expression and the role of IFI27 in IFN-elicited anti-hepatitis B virus (HBV) response in human hepatoma cells. **(A–D)** HepG2 and Huh7 cells were treated with IFN-α (100 ng/ml) and incubated for 0, 6, and 12 h. **(A,B)** The treated HepG2 and Huh7 cells were subjected to western blotting or immunoblotting using an anti-IFI27 antibody, where β-actin was taken as a normal internal reference control (bottom panels). **(C,D)** The total HBV RNA was isolated from the treated cells, and the expression of IFI27 of mRNA was determined by using qRT-PCR. Glyceraldehyde-3-phosphate dehydrogenase (GAPDH) mRNA expression was used as a loading control to normalized the data. **(E–G)** pCAGGS-HA-IFI27 or pCAGGS (empty vector) plasmid was transfected into HepG2.2.15. After 24 h of incubation, the cells were treated with IFN-α (100 ng/ml). **(E,F)** The relative expression level of IFI27 mRNA and total HBV mRNA was examined by qRT-PCR. GAPDH was used as an internal control. **(G)** HBV intracellular DNA expression level was determined by qPCR. *P ≤ 0.05, **P ≤ 0.01, ***P ≤ 0.001, ****P ≤ 0.0001.

*IFI27* or *ISG12a* is one of the most highly induced genes following treatment of cells with type I IFNs (Rosebeck and Leaman, 2008). To confirm this speculation, we cultivated HepG2 and Huh7 cells separately in a 12-well plate for 24 h and then treated them with IFN-α (100 ng/ml) in a time-dependent manner (0, 6, and 12 h). The samples were collected at indicated time points. The protein levels of IFI27 in both types of cell lines were examined with western blotting (Figures 1A,B). The total RNA was extracted from the cells, and the levels of mRNA of IFI27 were determined by qRT-PCR (Figures 1C,D). As expected, IFI27 was significantly upregulated in HepG2 and Huh7 following IFN-α treatment. The results revealed that IFI27 is one of the endogenous IFN-α-inducible genes in liver cells and may be involved in the molecular mechanism of IFN-α-mediated restriction of HBV.

Next, to judge the physiological role of IFI27 in IFN-elicited anti-HBV response, we use HepG2.2.15 cells, which are derived from the human hepatoblastoma cell line HepG2 and are characterized by having stable HBV expression and replication in the culture system (Sells et al., 1987). As a cell source, HepG2.2.15 cells can stably express HBV and these cells have been frequently used in studies of HBV infection assays (Zhao et al., 2011). The HepG2.2.15 cells were transfected with pCAGGS-HA-IFI27 plasmid or empty vector. After 24 h of incubation, the cells were treated with 100 ng/ml IFN-α. The expression levels of IFI27 mRNA were analyzed by qRT-PCR (Figure 1E). The total HBV mRNAs were efficiently reduced by IFN-α-treated cells as revealed by qRT-PCR (Figure 1F). As a result of the upregulation of IFI27, HBV intracellular DNA expression levels were also reduced as determined by qPCR (Figure 1G). These results indicate that following successful transfection, IFI27 potentiated the anti-HBV activity upon IFN-α treatment in HepG2.2.15 cells.

### Overexpression of IFI27 Inhibits HBV Gene Expression and Replication in HepG2 Cells

**FIGURE 2:**
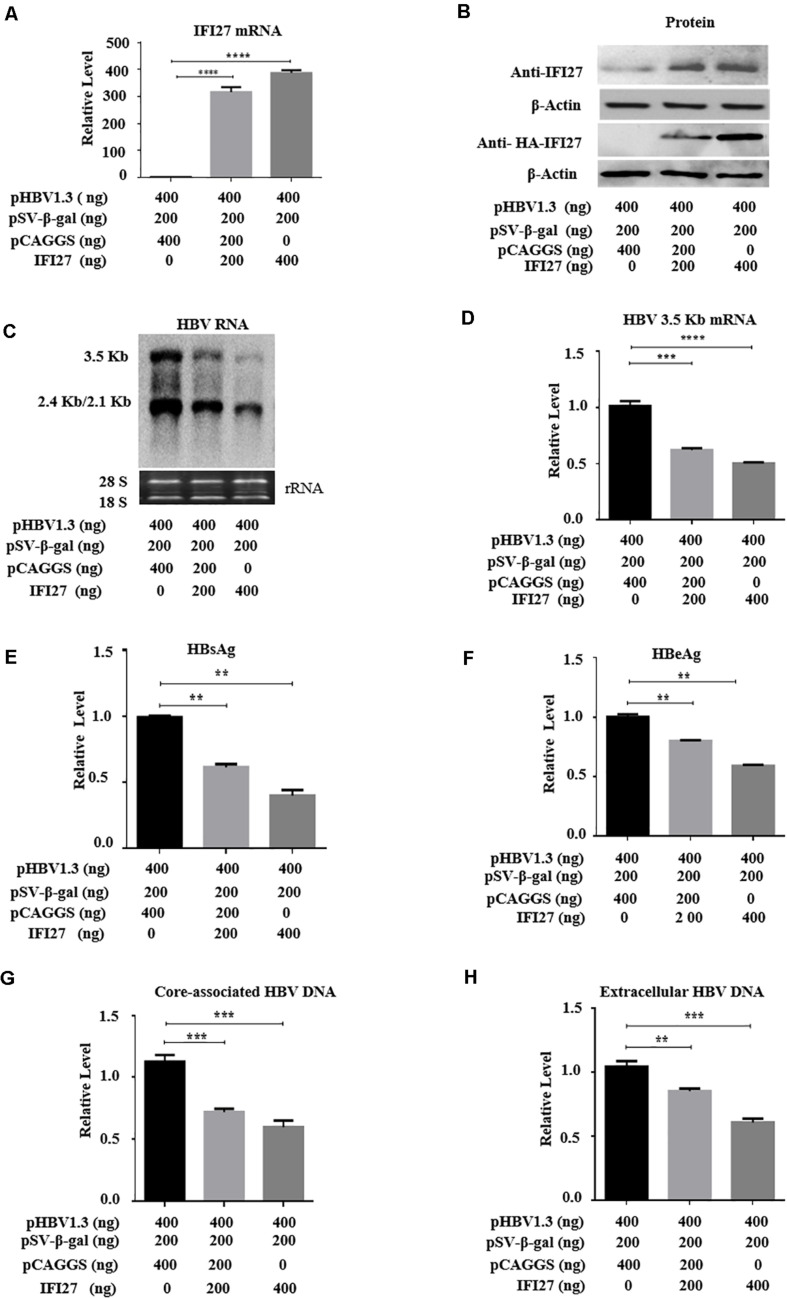
Overexpression of IFI27 inhibits HBV gene expression in HepG2 cells. **(A–F)** Human hepatoma cells (HepG2) were co-transfected with pHBV1.3 plasmid, pSV-β-gal, and pCAGGS-HA-IFI27 or pCAGGS (empty vector) at different concentrations for 48 h, as indicated in the artwork. **(A)** The IFI27 mRNA expression was detected by qRT-PCR. GAPDH was used as an internal control. **(B)** The expression levels of IFI27 protein were determined by western blotting using anti-IFI27 and anti-HA antibodies, respectively. β-Actin expression was used as a loading control. **(C)** The total HBV RNA was extracted and subjected to northern blot analysis. The rRNAs (28S and 18S) were used as the internal control. **(D)** Intercellular HBV 3.2 kb mRNA was detected by qRT-PCR. GAPDH mRNA expression was used to normalized the data. **(E,F)** Secreted HBsAg **(E)** and HBeAg **(F)** in the supernatants were determined by using ELISA. **(G,H)** HepG2 cells were transfected with pHBV1.3 plasmid vector together with pCAGGS-IFI27 or pCAGGS (empty vector) in a dose-dependent manner for 96 h. **(G)** The HBV intracellular core-associated and extracellular HBV DNA **(H)** from the supernatant were extracted and measured by qPCR. **P ≤ 0.01, ***P ≤ 0.001, ****P ≤ 0.0001.

The inhibitory effect of IFI27 on HBV replication has not been described, and we initially examined the role of IFI27 in HBV gene expression and replication. HepG2 cells were co-transfected with an HBV plasmid (pHBV1.3) and pSV-β-gal along with different doses of IFI27 expression vector (pCAGGS-HA-IFI27). The expression levels of IFI27 mRNA and protein were analyzed by qRT-PCR and western blotting, respectively (Figures 2A,B). The HBV RNA transcripts (3.5, 2.4, and 2.1 kb) were subjected to northern blot. The 3.5-kb transcript containing the pgRNA and the longer precore mRNAs were decreased remarkably upon IFI27 overexpression (Figure 2C). Besides, with the reduction of HBV RNAs, qRT-PCR indicated that the overexpression of IFI27 decreases the levels of HBV 3.2 kb mRNA significantly (Figure 2D). We observe the inhibitory effect of IFI27 against HBV replication by further measuring HBsAg and HBeAg in cell culture supernatant using ELISA (Figures 2E,F). We also illustrate the suppressive role of IFI27 on HBV gene expression and replication, and the core-associated HBV DNA in transfected HepG2 cells and HBV DNA (secreted) in the culture medium were extracted and observe by qPCR (Figures 2G,H). Both HBV intracellular core-associated and extracellular DNAs were highly restricted upon overexpression. Hence, our data suggested that IFI27 plays an inhibitory role in HBV gene expression and replication.

### Knockdown of IFI27 Enhances HBV Replication and Gene Expression in HepG2 Cells

**FIGURE 3:**
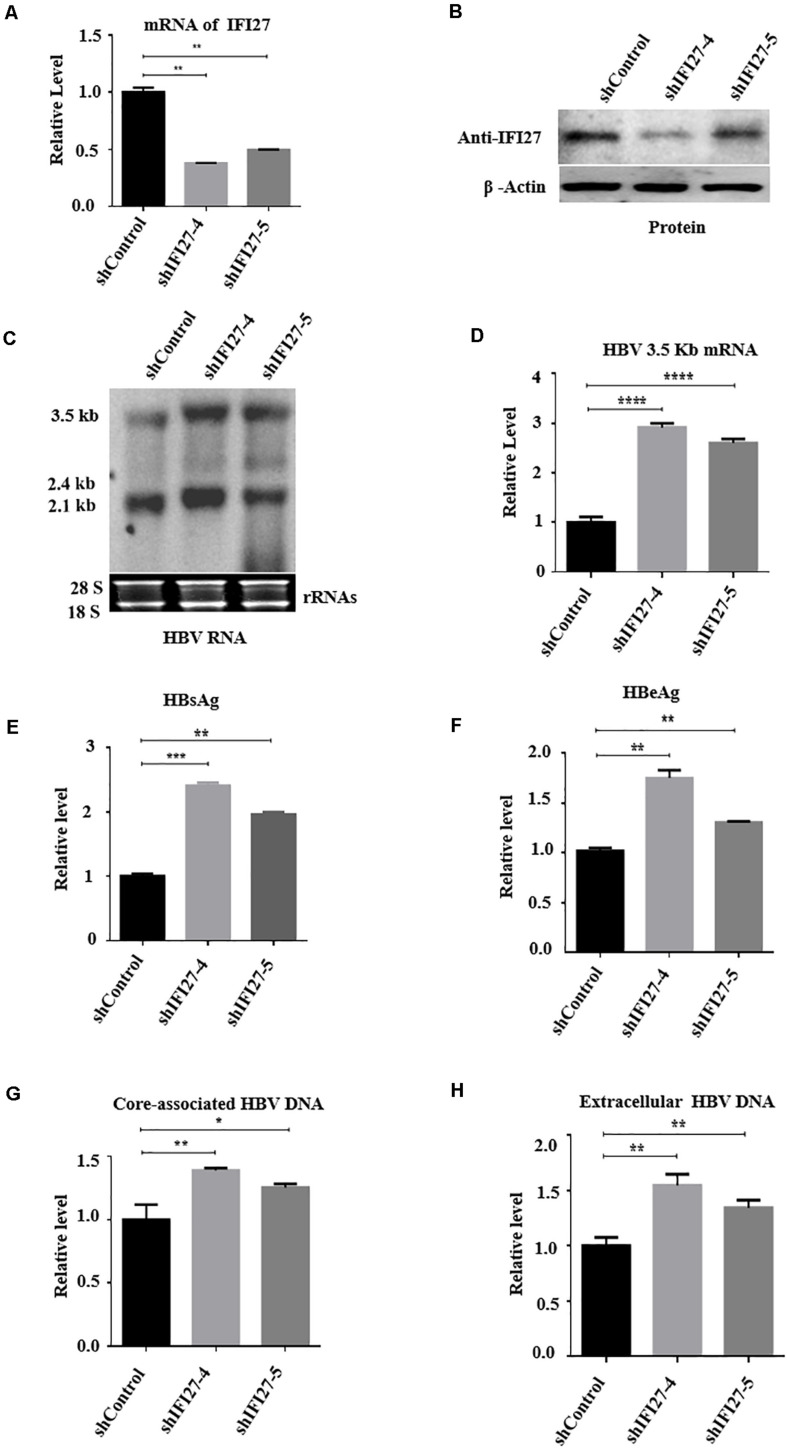
Knockdown of IFI27 enhances HBV gene expression in HepG2 cells. **(A–F)** HepG2 cells in a 12-well plate were transfected with HBV1.3 (800 ng) and pSV-β-gal (200 ng), and 24 h later, the cells were transduced with lentivirus expressing IFI27-targeting shRNAs (shIFI27-4 and shIFI27-5) or scrambled control (shControl). The lentivirus-transduced cells were collected after 3 days post-transduction. **(A)** The levels of IFI27 mRNAs were determined by qRT-PCR. Data were normalized to GPADH mRNA expression. **(B)** Expression levels of IFI27 protein were analyzed by western blotting using an IFI27-specific antibody. β-Actin was used as a loading control. **(C)** HBV total RNA from transducing cells was extracted and HBV transcripts were analyzed by northern blot. The 28S and 18S rRNAs were used as an internal control. **(D)** The HBV 3.2-kb mRNA was subjected to qRT-PCR. GAPDH mRNA expression was used as a loading control. **(E,F)** Expression of secreted HBsAg **(E)** and HBeAg **(F)** was detected using ELISA. **(G,H)** The HBV intracellular core-associated **(G)** and extracellular HBV DNA **(H)** from the supernatant were extracted 4 days post-transduction and subjected to qPCR. *P ≤ 0.05, **P ≤ 0.01, ***P ≤ 0.001, ****P ≤ 0.0001.

To validate further the inhibition role of IFI27 on HBV gene expression, we performed gene knockdown or silencing in HepG2 cells. For knockdown, the IFI27 expression in HepG2 cells was downregulated by transducing with shRNA-lentiviral vectors such as shIFI27-4 and shIFI27-5 or scramble control (shControl), targeting IFI27. HepG2 cells were transfected with pHBV1.3 and pSV-β-gal plasmids and incubated for 24 h. After 24 h, the medium was discarded and the cells were transduced with shIFI27-4 and shIFI27-5 or scramble control (shGFP) and incubated for further 48 h. The two independent shRNAs, shIFI27-4 and shIFI27-5, showed strong IFI27 knockdown effects in the cells. The levels of IFI27 mRNA were examined by qRT-PCR (Figure 3A), and western blotting confirmed the expression of IFI27 protein (Figure 3B). Both mRNA and protein expression levels of IFI27 were significantly suppressed as compared with the scramble control, which indicates that shIFI27-4 and shIFI27-4 are effective. The HBV transcripts (3.5, 2.4, and 2.1 kb) in transduced HepG2 cells were evaluated through northern blot (Figure 3C). Here, we also investigated whether IFI27 affects the level of HBV 3.2 kb mRNA, and the qRT-PCR result revealed that the HBV 3.2-kb mRNA levels increased significantly upon silencing of IFI27 (Figure 3D). Concordantly, knockdown of IFI27 in HepG2 cells also enhanced the secretion of HBsAg and HBeAg proteins in the culture supernatant, as confirmed by ELISA (Figures 3E,F). As a result of the downregulation of IFI27, the levels of HBV intracellular core-associated and extracellular HBV DNA were also increased, as determined by qPCR (Figures 3G,H). Overall, these results suggested that the silencing of IFI27 significantly enhanced HBV gene expression in hepatocytes.

### IFI27 Knockout (IFI27-KO) in HepG2 Cells Promote HBV Replication

**FIGURE 4:**
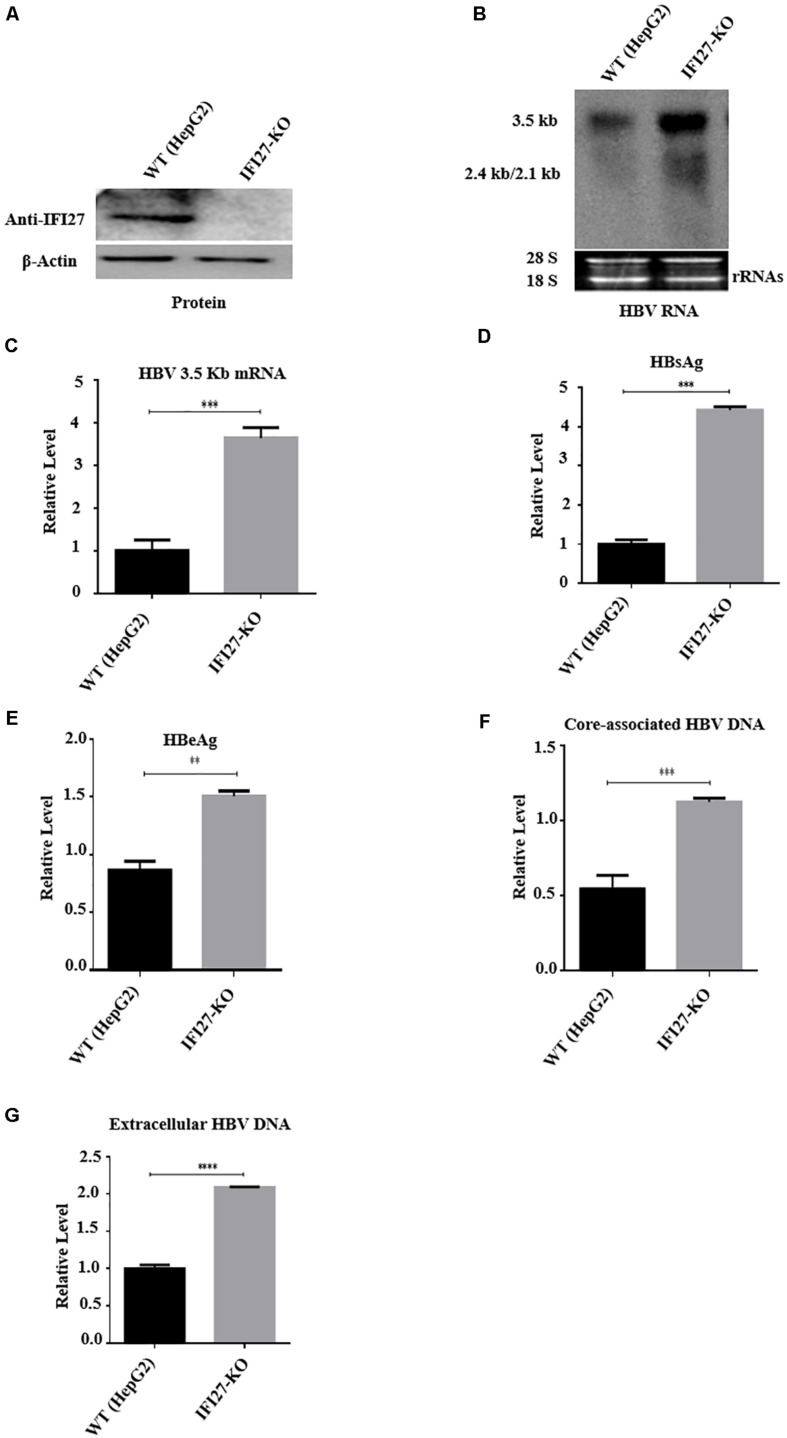
IFI27 knockout (IFI27-KO) in HepG2 cells promotes HBV replication. **(A–F)** IFI27-deficient HepG2 cell line (HepG2 IFI27-KO) was generated by the CRISPR/Cas9 system using a guide RNA; the sequence is already shown in the methodology. HepG2 parent cells (wild-type) and the IFI27 knockout HepG2 cell line were transfected with HBV1.3 (800 ng) and pSV-β-gal (200 ng) and harvested after 48 h. **(A)** Western blot analysis was used to confirm IFI27 protein expression using an anti-IFI27 antibody and β-actin was used as a loading control. **(B)** Total RNA from HepG2 parent cell and IFI27-KO cells was extracted and detected with northern blotting. The 28S and 18S rRNAs were used as the internal control. **(C)** The HBV 3.2-kb mRNA was separately subjected to qRT-PCR. GAPDH was used as a loading control. **(D,E)** The secreted HBsAg **(E)** and HBeAg **(F)** proteins were analyzed using ELISA. **(F,G)** HepG2 parent cells and the IFI27 knockout HepG2 cell line were transfected with pHBV1.3 and harvested after 96 h. The HBV intracellular core-associated **(F)** and extracellular HBV DNA **(G)** from the supernatant were purified and subjected to qPCR. **P ≤ 0.01, ***P ≤ 0.001, ****P ≤ 0.0001.

Based on the results of overexpression and silencing of IFI27 on replication of HBV, we further confirm our results by using CRISPR/Cas9 gene-editing technology to generate a stable HepG2 cell line in which the *IFI27* gene was completely knocked out (HepG2 IFI27-KO). The HBV replication-competent plasmid (pHBV1.3) was transfected into HepG2 (wild-type) and HepG2 IFI27-KO cells. The suppression level of IFI27 protein was analyzed with western blotting (Figure 4A). These results suggested that the level of IFI27 protein expression was significantly reduced if we compare it with wild-type (parent cells), which determines the efficiency of knocking out of IFI27 in HepG2 cells. The northern blot result revealed that IFI27-KO HepG2 cells highly increased the level of HBV transcripts (2.5, 2.4, and 2.1 kb) (Figure 4B). In addition, the HBV 3.2-kb mRNA in HepG2 IFI27-KO cells more potently increased as revealed by qRT-PCR (Figure 4C). With the enhancement of HBV RNAs, as compared with WT cells, secreted HBsAg and HBeAg protein levels in the supernatant were efficiently enhanced in IFI27-KO HepG2 cells (Figures 4D,E). Our result showed that the level of HBV intracellular core-associated and extracellular DNA of HBV in supernatant was also increased in HepG2 IFI27-KO cells relative to parent cells as revealed by qPCR (Figures 4F,G). Collectively, these findings demonstrate that IFI27 knockout in hepatocytes increased HBV gene expression and replication.

### IFI27 Suppresses HBV Gene Expression by Inhibiting EnhII/Cp Promoter Activity

**FIGURE 5:**
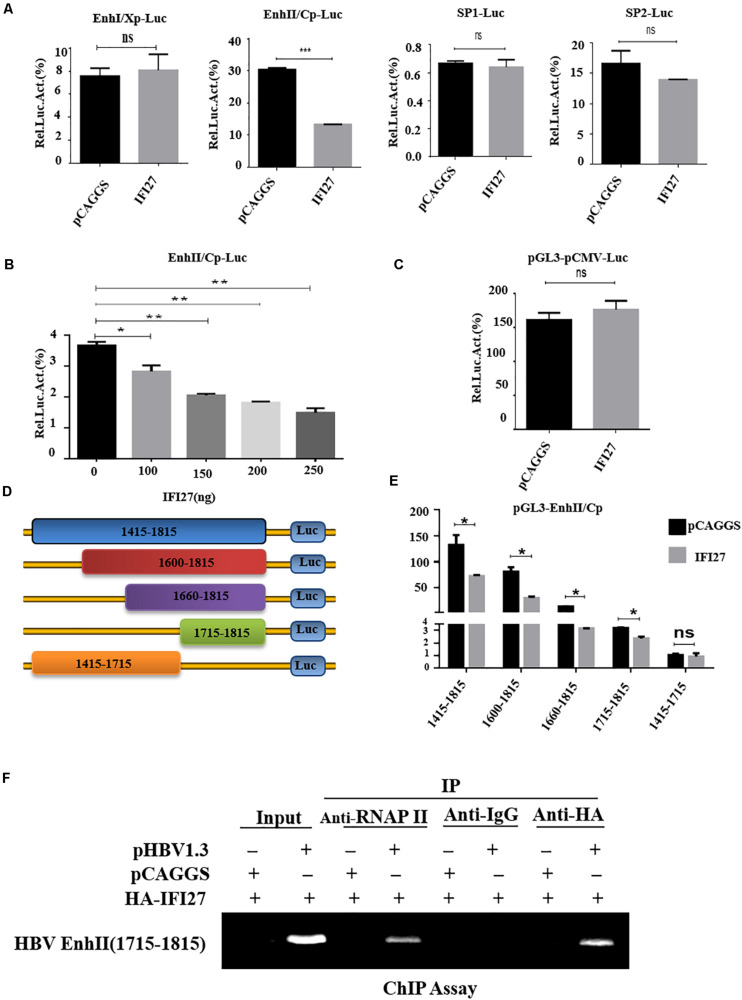
IFI27 represses HBV gene expression by inhibiting EnhII/Cp promoter activity. **(A)** HepG2 cells plated in a 24-well plate were co-transfected with reporter plasmids pGL3-EnhI/Xp, pGL3-EnhII/Cp, pGL3-SP1, and pGL3-SP2 (200 ng each) together with pCAGGS-HA-IFI27 or pCAGGS (250 ng). Luciferase activities of Firefly relative to Renilla were determined. pRL-TK (50 ng) was used as a control of transfection efficiency. **(B)** HepG2 cells were co-transfected with reporter plasmid pGL3-EnhII/Cp-Luc and/or pCAGGS-HA-IFI27 or pCAGGS at different concentrations for 2 days (48 h). Luciferase assay was measured and normalized with control (pRL-TK). **(C)** HepG2 cells were co-transfected with pGL3-pCMV-Luc (200 ng) along with pCAGGS-IFI27 or pCAGGS. Luciferase assay was measured and normalized with pRL-TK. **(D)** The schematic diagram of serial deletion mutants of the EnhII/Cp reporter. **(E)** IFI27 and their effect on serial deletion of HBV EnhII/Cp promoter. **(F)** HepG2 cells were seeded in 10 cm dish and then co-transfected with pHBV 1.3 (5 μg) and pCAGGS-HA-IFI27 or pCAGGS (5 μg) for 48 h. The CHIP assay was achieved using an anti-HA antibody to examine the binding capacity of IFI27 to EnhII/Cp. The amplification of extracted chromatin DNA was done by PCR and determined by agarose gel electrophoresis. Normal IgG is used as a negative control (IgG), while RNA polymerase II acts as a positive control (RNAPII) in this experiment. *P ≤ 0.05, **P ≤ 0.01, ***P ≤ 0.001 and ns (non-significant).

To examine the mechanism of IFI27-mediated HBV repression, the role of IFI27 in the regulation of HBV promoters were assessed. HepG2 cells were co-transfected with reporter plasmids pGL3-EnhI/Xp, pGL3-EnhII/Cp, pGL3-SP1, and pGL3-SP2 along with pCAGGS–IFI27 or pCAGGS. The results from the luciferase-based reporter assay revealed that EnhII/Cp activity was suppressed by IFI27, whereas IFI27 did not affect the activities of the EnhI/Xp, SP1, and SP2 promoters (Figure 5A). To confirm the suppressive effect of IFI27 in the inhibition of HBV EnhII/Cp promoter, HepG2 cells were co-transfected with pGL3-EnhII/Cp together with IFI27 expression plasmid or control plasmid at different concentrations. According to the reporter assay, the activity of EnhII/Cp was inhibited by IFI27 in a dose-dependent manner (Figure 5B). To further validate the specific inhibitory effect of IFI27 on HBV EnhII/Cp, we replaced EnhII/Cp with pCMV (cytomegalovirus promoter). HepG2 cells were co-transiently transfected with pGL3-pCMV plasmid along with IFI27 expression plasmid, or control plasmids were subjected to dual-luciferase activity. Importantly, IFI27 did not affect the activity of the pCMV promoter (Figure 5C). This result indicated that IFI27 is solely responsible for the inhibition of EnhII/Cp. We further examined the mechanisms of IFI27 inhibition on the HBV EnhII/Cp promoter and identified which region is targeted by IFI27. A series of EnhII/Cp deletion mutants were constructed and then these constructs were subcloned into the pGL3 basic vector to generate five reporters (Figure 5D). The deletion region (nt 1715–1815) of EnhII/Cp is reduced potently by IFI27. Hence, the region from nt 1715 to 1815 of HBV genome could be responsible for the suppressive effect of IFI27 (Figure 5E). Then, the study conducted a ChIP assay to investigate whether IFI27 could bind to the region from nt 1715 to 1815 in HBV EnhII/Cp promoter. Fragments of HBV EnhII/Cp promoter were detected in anti-HA-IFI27 antibody immunoprecipitated candidates in HepG2 cells transfected with pHBV1.3 plasmids, but were not detected in cells transfected with control vector (Figure 5F). Taken together, these data indicated that IFI27 binds to the EnhII/Cp region and displays an inhibitory effect on HBV replication and transcription.

### IFI27 Restricts HBV Gene Expression and Replication *in vivo*

**FIGURE 6:**
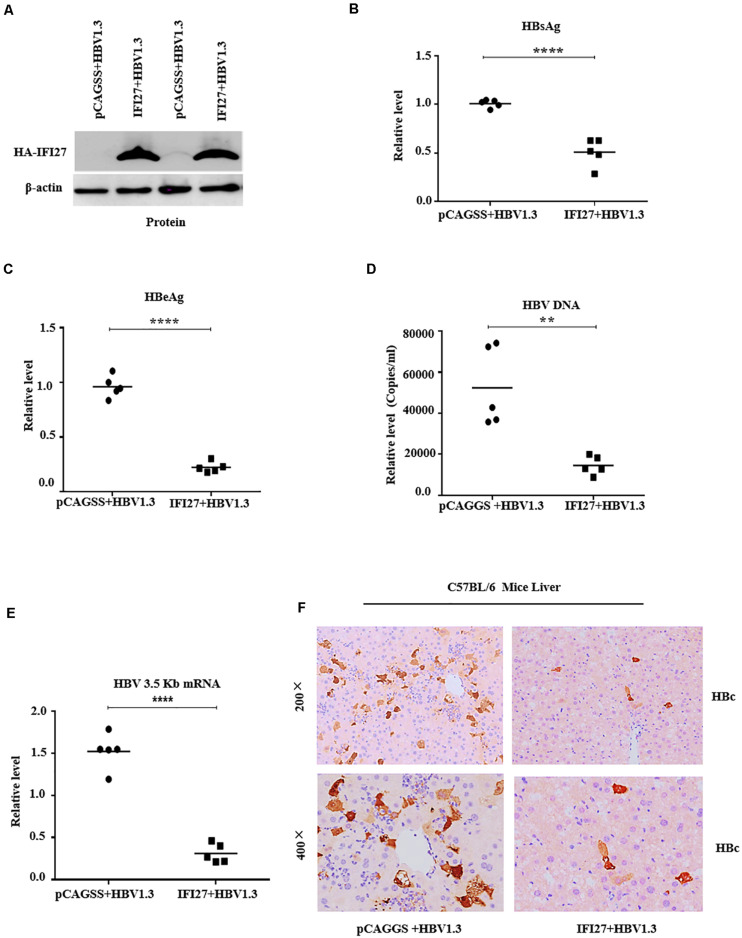
IFI27 suppresses HBV replication and gene expression *in vivo.* **(A–F)** Two groups of C57BL/6 mice (*n* = 10) were co-injected with pHBV1.3 along with pCAGGS-HA-IFI27 or pCAGGS. After 4 days of hydrodynamic injection, the mice were sacrificed and the livers and blood were processed for analyses. **(A)** The expression level of IFI27 was determined by western blot using anti-HA antibodies, and the levels of β-actin serve as loading controls (lower panel). **(B,C)** The titer of secreted HBsAg **(B)** and HBeAg **(C)** proteins expressed in mice blood was measured using ELISA. **(D)** The level of HBV DNA from sera was also evaluated using qPCR. **(E)** Total HBV RNA was isolated from the liver tissues, and the levels of HBV pgRNA were measured by qRT-PCR. The level of mice GAPDH (mGAPDH) was used as an internal control. **(F)** Immunohistochemical staining was used to determine the HBcAg levels in the liver. **P ≤ 0.01, ****P ≤ 0.0001.

Because IFI27 was shown to restrict HBV gene expression and replication in HepG2 cells, we also investigated the role of IFI27 expression on HBV replication in the mouse model system *in vivo*. C57BL/6 male mice were co-delivered with pHBV1.3 and pSV-β-gal, together with pCAGGS-HA-IFI27 plasmids or control vector (pCAGGS) through hydrodynamic injection and kept for 4 days. After scarifying all the mice, the effect of IFI27 on HBV replication was assessed. The IFI27 expression level was detected with western blot. The result showed a high expression of IFI27 protein level in mice liver (Figure 6A). In the sera of treated mice, HBsAg, HBeAg, and HBV DNA were tested. The levels of HBsAg and HBeAg were measured by ELISA (Figures 6B,C) and HBV DNA was evaluated through qPCR (Figure 6D). These data suggested that the expression of HBsAg and HBeAg and HBV DNA were remarkably diminished *via* IFI27 expression. To further validate HBV transcription *in vivo*, HBV 3.5 kb mRNA levels were determined by qRT-PCR (Figure 6E), which was significantly suppressed by IFI27. Moreover, the immunohistochemical (IHC) staining of the mice liver tissues reveals that the HBV core proteins (HBc) were reduced effectively in the presence of IFI27 (Figure 6F). Together, these data indicated that IFI27 has an inhibitory effect on HBV transcription and replication *in vivo.*

## Discussion

**FIGURE 7:**
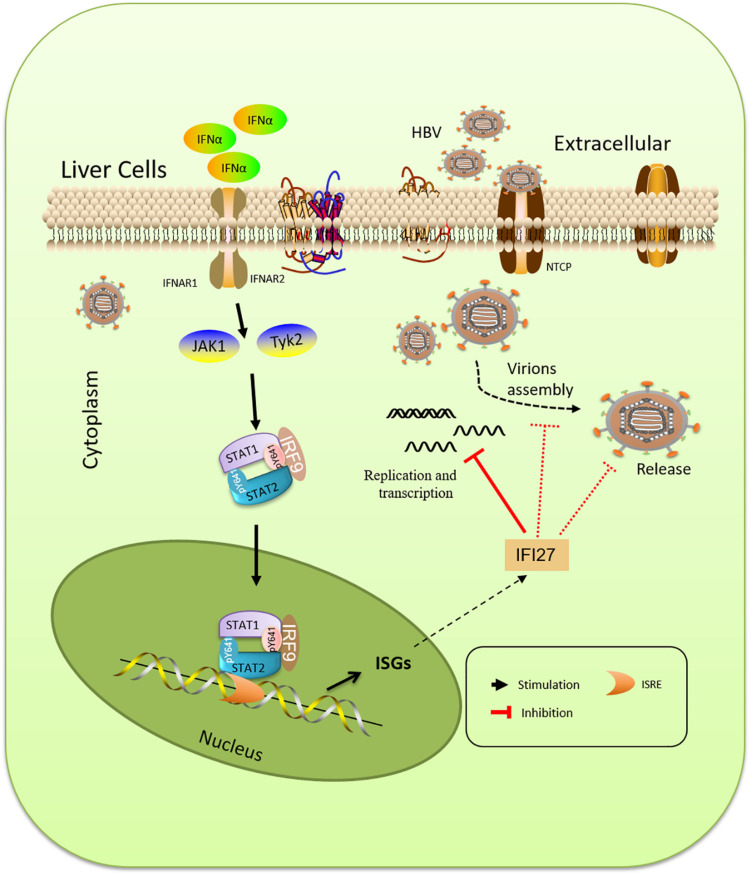
Schematic representation of IFN-α and IFN-stimulated gene (ISG) induction pathway. The type I IFN (IFN-α) is cascaded *via* the Janus kinase (JAK)/signal transducer and activator of transcription (STAT) pathway, resulting in the expression of a large spectrum of activated transcriptional ISGs. IFI27 is an ISG that suppresses viral gene expression, prevents transcript production, and decreases the accumulation of viral replicative intermediates.

Interferons are a set of signaling proteins produced and released by host cells against a variety of pathogens (De Andrea et al., 2002; Lee and Baldridge, 2017). IFN-α, a type I IFN produced by lymphocytes, is one of the naturally occurring cytokines with immunomodulatory and antiviral mechanisms (Peters, 1996). In terms of mechanism, Kupffer cells of the liver identify HBV constituents by its Toll-like receptors (TLRs) and RIG-1-like receptors (RLRs) and induce the production of type I IFN, which directly restrict HBV or exert an immunoregulatory function, which is the key point of anti-HBV innate immunity (Seki et al., 2000). In addition, type I IFN can activate other important members of innate immunity (Kadowaki and Liu, 2002), such as natural killer (NK) cells and natural killer T (NKT) cells, recruit them to the infected tissue, and recognize infected hepatocytes. NK cells directly kill the infected hepatocytes through the killer-cell immunoglobulin-like receptor system (Chen et al., 2005). Antiviral activities include degradation of viral mRNA, inhibition of viral protein synthesis, and prevention of infection of cells (Rijckborst and Janssen, 2010). Though IFN is largely used in clinical settings for the treatment of chronic HBV, the underlying mechanism of its antiviral effects is still unclear (Tan et al., 2019). Due to non-oral, severe side effects and limited efficacy in the suppression of HBV DNA replication, IFN-α is not a first-line therapy drug suggested by the guidelines and has not been frequently used in the clinic (Zhuang, 2012). The pegylated IFN-α (peg-IFN-α-2a and α-2b) are existing treatments for HBV therapy (Tang et al., 2018). IFN-α induces more than 300 ISGs, which acts as an innate immune effector to take part in antiviral processes (Schoggins and Rice, 2011). As it is already mentioned in Figure 7, the antiviral processes of IFN-α is cascaded via Janus kinase (JAK)/signal transducer and activator of transcription (STAT) pathway, as a result of these ISGs. In a previous study, it was reported that TRIM22, which is an ISG expressed in response to IFNs, displayed anti-HBV activity both *in vitro* and *in vivo* (Gao et al., 2009). Type I IFNs induce TRIM25 through an IL-27-dependent manner to suppress HBV replication (Tan et al., 2018). APOBEC3A/B was shown to play a key role in the degradation of nuclear HBV cccDNA (Labrada et al., 2002; Lucifora et al., 2014). Other ISGs such as PVRL4, TRIM38, GBP2, TRIM5g, CBFb, and Gadd45g interact with HBx of HBV and inhibit their replication (Carter et al., 2005). In the analysis of intrahepatic ISGs and to assess the role of IFI27 concerning HBV immune response, we found that IF27 was upregulated in HepG2 and Huh7 cells in a time-dependent manner when treated with IFN-α. Furthermore, we also evaluated that IFN-α-induced IFI27 is responsible for IFNα-mediated suppression of HBV (Figures 1E–G). For this purpose, we used HepG2.2.15 cells, which were stably transduced with the HBV genome and the best source for HBV infection assays. It was reported that HepG2.2.15 cells are interesting tools for the screening of antiviral molecules (van de Klundert et al., 2016).

In a previous report, IFI27 was first proposed to have antiviral activity against Sindbis virus infection in mice (Labrada et al., 2002) that can also reduce infection of hepatitis C (Itsui et al., 2006), though the basic mechanisms remain unidentified. Also, ectopic expression of IFI27 inhibits hepatitis C virus replication in both HCV infectious culture systems and replicon cells (Xue et al., 2016). In contrast to a previous study that IFI27 has antiviral activity, we hypothesized the role and antiviral mechanism of the *IFI27* gene against HBV. We found that IFI27 inhibits HBV replication and gene expression *in vitro*. The expression levels of HBV DNA, proteins, and RNA transcripts were reduced by the overexpression of IFI27 and increased by the downregulation of IFI27 (Figures 2, 3). Moreover, the knocking out of IFI27 vigorously enhanced the HBV replication in HepG2 cells (Figure 4). The inhibitory role of IFI27 in HBV gene expression and replication was also confirmed *in vivo*. C57BL/6 mice were used, which are the most suitable laboratory animal for such immunological research. The overexpression of IFI27 remarkably reduced the HBV proteins, DNA, and RNA in the mouse sera and liver tissues (Figure 6). The overexpression, knockdown, and knockout analysis clearly revealed the anti-HBV effect of IFI27, but the level of HBsAg was dramatically inhibited in the overexpression and enhanced in the knockdown and knockout of IFI27, respectively, indicating that some other factors or post-transcriptional mechanism might be involved as IFI27 has no effect on SP1 and SP2 activities.

Based on a previous report, it was discovered that an interferon-stimulated gene ISG20 has antiviral activity against HBV, binds to its EnhII/Cp region, and regulates HBV at the transcriptional level (Park et al., 2020). It was revealed that an ISG TRIM22 significantly inhibited the activity of HBV and EnhII/Cp promoter, which plays an essential role in HBV replication (Gao et al., 2009). Consistent with the previous report, we demonstrate that IFI27 suppressed the activity of EnhII/Cp promoter while there was no effect of IFI27 on other regulatory elements of HBV (SP1, SP2, and EnhI) as revealed by dual-luciferase reporter assays (Figure 5A). Further dual-luciferase reporter assay revealed that IFI27 does not affect the activity of pGL3-pCMV constructs, which proves the specificity of the IFI27-mediated HBV inhibition through the EnhII/Cp promoter (Figure 5C). A detailed analysis indicated that the region of the HBV genome from nt 1715 to 1815 played a role in IFI27-mediated EnhII/Cp promoter inhibition (Figures 5E,F). Thus, we proposed that IFI27 targeting the EnhII/Cp region leads to the restriction of HBV gene expression and replication.

We concluded from the above facts that IFI27 has an antiviral effect on HBV gene expression and replication *in vitro* and *in vivo*. We showed that IFI27 is highly expressed in human hepatoma cell lines by the induction of IFN-α. The overexpression of IFI27 inhibited HBV replication and gene expression, while the knockdown and knocking out of IFI27 in HepG2 cells enhanced HBV replication and gene expression. A further study reveals that IFI27 restricts HBV replication through directly binding to EnhII/Cp DNA and suppressing its activity. Taken together, our data further recommended understanding the antiviral activity of IFI27. The purpose of IFI27 combined with the cellular immune system needs to be further characterized in HBV infection.

## Data Availability Statement

The original contributions presented in the study are included in the article/supplementary material, further inquiries can be directed to the corresponding author/s.

## Ethics Statement

The animal study was reviewed and approved by the Institutional Animal Care and Use Committee of Wuhan University.

## Author Contributions

LZ, LMZ, and HU conceived the study and designed the experiments. HU, MS, and KU performed the experiments. MH, JF, DG, and RH assisted with the experiments. HU, MS, MAS, QL, and TX analyzed the data. HU wrote the initial draft of the manuscript. LZ and YC revised the manuscript. All others read and commented on the manuscript.

## Conflict of Interest

The authors declare that the research was conducted in the absence of any commercial or financial relationships that could be construed as a potential conflict of interest.
